# The influence of the Pringle maneuver in laparoscopic hepatectomy: continuous monitor of hemodynamic change can predict the perioperatively physiological reservation

**DOI:** 10.3389/fdata.2023.1042516

**Published:** 2023-06-14

**Authors:** Yi-Chan Chen, Min-Hsuan Lee, Shan-Ni Hsueh, Chien-Liang Liu, Chung-Kun Hui, Ruey-Shyang Soong

**Affiliations:** ^1^Department of General Surgery, Keelung Chang Gung Memorial Hospital, Keelung, Taiwan; ^2^College of Medicine, Chang Gung University, Taoyuan, Taiwan; ^3^Department of Industrial Engineering and Management, National Yang Ming Chiao Tung University, Hsinchu, Taiwan; ^4^Department of Anestheiology, Keelung Chang Gung Memorial Hospital, Keelung, Taiwan; ^5^Division of Transplantation, Department of Surgery, Taipei Municipal Wan-Fang Hospital, Taipei Medical University, Taipei, Taiwan; ^6^College of Medicine, Taipei, Medical University, Taipei, Taiwan

**Keywords:** hepatocellular carcinoma, Pringle maneuver, laparoscopic hepatectomy, goal directed fluid management, FloTrac

## Abstract

**Importance:**

This is the first study to investigate the correlation between intra-operative hemodynamic changes and postoperative physiological status.

**Objective:**

**Design, settings, and participants:**

Patients receiving laparoscopic hepatectomy were routinely monitored using FloTract for goal-directed fluid management. The Pringle maneuver was routinely performed during parenchymal dissection and the hemodynamic changes were prospectively recorded. We retrospectively analyzed the continuous hemodynamic data from FloTrac to compare with postoperative physiological outcomes.

**Exposure:**

The Pringle maneuver during laparoscopic hepatectomy.

**Main outcome(s) and measure(s):**

**Results:**

Stroke volume variation that did not recover from the relief of the Pringle maneuver during the last application of Pringle maneuver predicted elevated postoperative MELD-Na scores.

**Conclusions and relevance:**

The complexity of the hemodynamic data recorded by the FloTrac system during the Pringle Maneuver in laparoscopic hepatectomy can be effectively analyzed using the growth mixture modeling (GMM) method. The results can potentially predict the risk of short-term liver function deterioration.

## Introduction

The multidisciplinary treatment of liver cancer, regardless of whether it is primary or secondary, is the consensus in the modern era. In multiple treatments, liver resection provides better survival rates (Xu et al., 2018). However, liver transection has been related to torrential bleeding due to the abundant vascular system, including the inflow system (hepatic artery, portal hepatis) and outflow system (hepatic vein), especially in cirrhotic or fatty livers (Jarnagin et al., 2002). The excess bleeding is related to postoperative decompensations of liver function, and massive blood transfusions lead to inferior survival outcomes. Even with advanced surgical techniques and perioperative care, the reported postoperative complicated can be up to 40%, with 1 to 5% mortality (Melloul et al., 2016). The Pringle maneuver was developed by James Hogarth Pringle in the early 1900s (Pringle, 1908) and has been adapted to hepatectomy commonly to reduce intraoperative bleeding during parenchymal transections. Previous prospective studies have shown the safety and efficacy of the intermittent Pringle maneuver during hepatectomy if the total ischemic time is within 120 mins (Man et al., 1997). Postoperative liver failure has not been observed with limited use of the Pringle maneuver, and liver function can recover with adequate perioperative care.

Laparoscopic hepatectomy was first performed in 1993 (Kaneko et al., 1996) and the indications of laparoscopic hepatectomy have grown widely in recent decades to treat liver tumors, including benign lesions, hepatocellular carcinoma (HCC), intrahepatic cholangiocarcinoma, and metastatic malignancy mainly from colorectal cancer (Buell et al., 2009). A combination of the Pringle maneuver, a lower central venous pressure (CVP) level of 5mmH_2_O, and keeping pneumoperitoneal pressure at 10–15 mmH_2_O helps to reduce intraoperative bleeding during laparoscopic hepatectomy (Liu et al., 2021).

Goal-directed fluid management under the guidance of invasive monitoring during major operations has been shown to be an effective and safe strategy to reduce excess fluid transfusion and is suggested in the protocols of enhanced recovery after surgery (ERAS) (Kendrick et al., 2019). Continuous monitoring of cardiac output (CO) and stroke volume variation (SVV) is crucial to maintain stable hemodynamics during major operations. The pneumoperitoneum and low CVP level are the two main factors that compromise the stability of hemodynamic activity. The FloTrac™ device uses arterial pressure waveform analysis to determine CO. It comprises a special transducer that attaches to an existing arterial cannula and then connects to a processing/display unit. CO is calculated from an arterial pressure-based algorithm that utilizes the relationship between pulse pressure and stroke volume. The dynamic changes of CO, SVV, systemic vascular resistance (SVR), and systemic vascular resistance index (SVRI) during an entire procedure is continuously recorded every 20 seconds. All data are automatically recorded and can represent the actual physiological changes during the entire procedure. Although previous studies have proved the safety of the intermittent Pringle maneuver (Man et al., 1997; Cannistrà et al., 2016), short-period organ insufficiency is still encountered after repeated ischemic reperfusion injury. Even under the goal-directed fluid management protocol, previous studies have shown heterogenous results that the given intravenous fluids decreased but the perioperative complications and length of hospital stay did not remarkably decrease (Correa-Gallego et al., 2015; Weinberg et al., 2019a,b; Jongerius et al., 2021). However, one recent observation study for goal-directed fluid management in laparoscopic hepatectomies revealed a lower incidence of postoperative acute kidney injury under goal-directed fluid management (Imai et al., 2022).

### Research gap

There was no dynamic real-time data with which to study the physiological changes under the Pringle maneuver. All the previous studies were conducted in the open hepatectomy era. Goal-directed fluid therapy in open hepatectomies was also studied with heterogenous outcomes, and a recent study to investigate goal-directed fluid therapy revealed that it can be helpful in preventing postoperative acute kidney injury, but no data was found in the literature that addressed the physiologic changes under minimally invasive surgical conditions. In this study, we used the growth mixture modeling (GMM) method to analyze the continuous data recorded by the FloTrac system to investigate the correlation between intraoperative physiological changes caused by ischemic reperfusion of the liver parenchyma in laparoscopic hepatectomies and the dynamic postoperative physiological changes. To the best of our knowledge, this is the first study to investigate the correlation between real-time hemodynamic and postoperative physiological changes.

## Methods

### Patients and methods

We prospectively enrolled patients undergoing laparoscopic hepatectomies from July 2019 to June 2021. The etiologies for hepatectomy include hepatocellular carcinoma, cholangiocarcinoma, colorectal cancer with liver metastasis, and benign tumors. Preoperative basic liver function include coagulation test and indocyanine green (ICG) test to determine further reserved liver function after liver resection.

### Perioperative management

**Figure 1:**
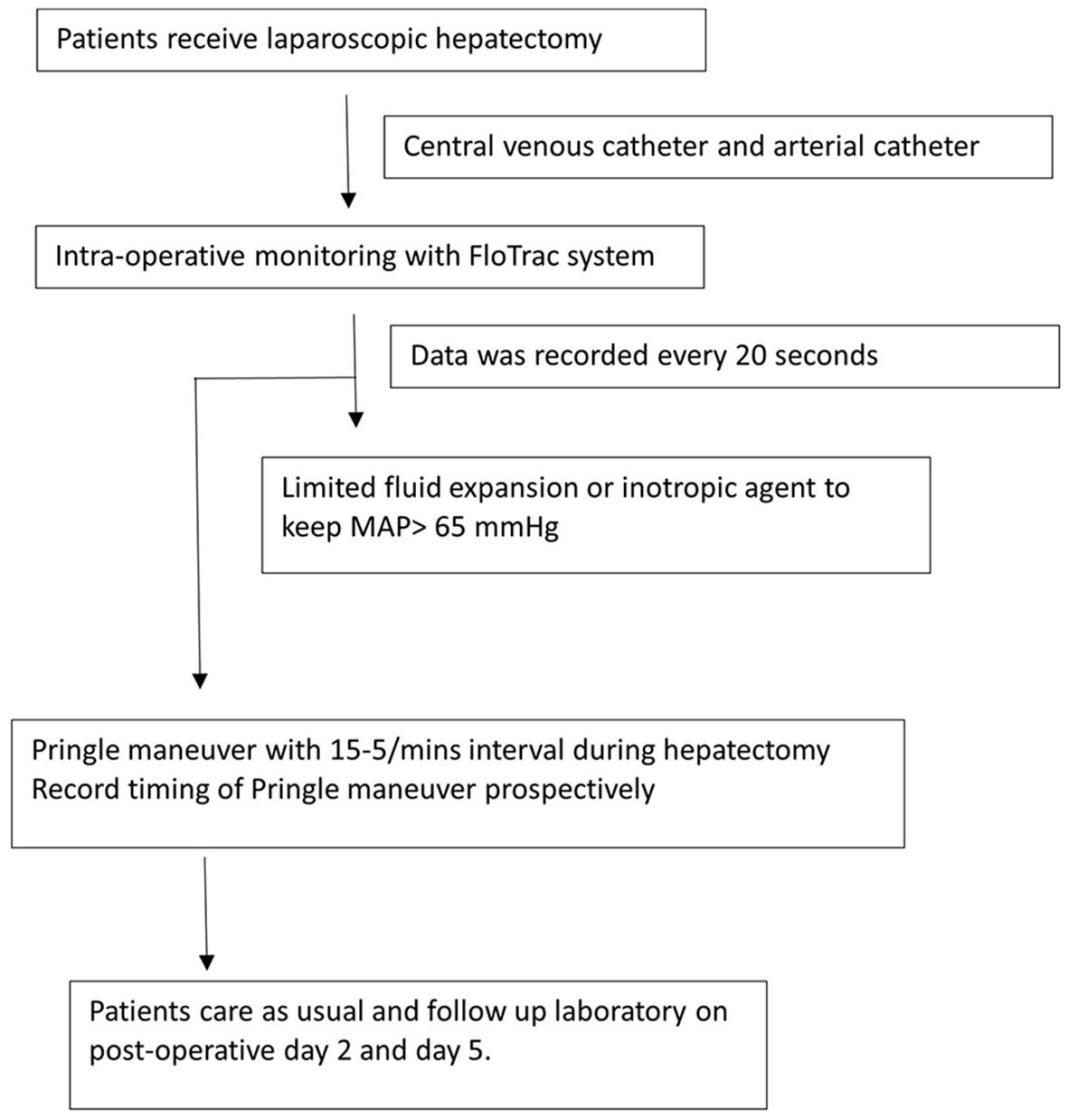
Perioperative management of the patients underwent laparoscopic hepatectomy under the monitoring of FloTrac system. MAP, mean arterial pressure.

All patients received limited fluid infusion before the operation to keep the CVP level low. No routine preoperative colon preparation was prescribed. In the operating room, a central venous catheter, arterial catheter, continuous electrocardiograms, Foley catheter, FloTrac system, and pulse oximeter were employed routinely. An inotropic agent was prescribed to keep mean arterial pressure (MAP) > 65 mmHg intraoperatively with limited volume infusion. If massive blood loss was encountered, blood transfusion with red blood cells or plasma would be prescribed to keep MAP > 65 mmHg after a team discussion by the anesthesiologist and surgeons. The algorithm of the perioperative management is showed in Figure 1.

### Surgical procedure

All surgeries were performed by a liver team with two fixed surgeons and a corresponding anesthesiologist for general anesthesia. The central venous catheter (CVP) and arterial catheter were inserted after anesthesia and connected to the Flotrac system. Hepatic parenchymal resection was performed by crush–clamp method using a Harmonic Hi10000 (Ethicon Endo-Surgery, Cincinnati, OH, USA), and Cavitron ultrasonic surgical aspirator (CUSA) together with a bipolar clamp coagulation system. We routinely used the Pringle maneuver with Huang's loop for inflow occlusion with 5-to-15 mins occlusion-releasing intervals.

#### Data collection and analysis

**Figure 2:**
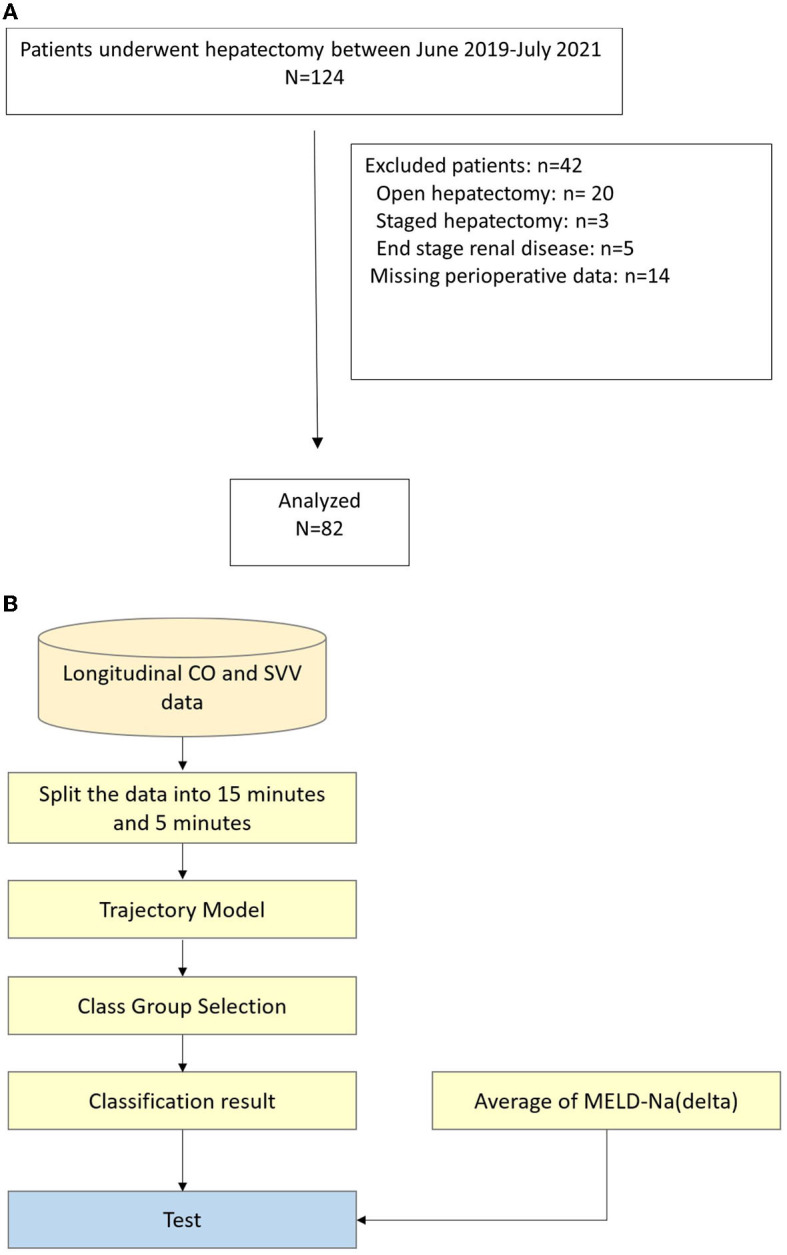
**(A)** Flow chart of patients selection process, **(B)** Flow chart of data analysis. CO, cardiac output; SVV, stroke volume variation; MELD-Na, model for end-stage liver disease-Na.

Of the 124 patients who underwent hepatectomy between June 2019 and July 2020, 82 were included. The reasons for exclusion were open hepatectomy, multiple metastatic tumors requiring staged hepatectomy, end stage renal disease before hepatectomy, and missing perioperative data including the FloTrac recording and postoperative laboratory examination as shown in Figure 2. All operations were performed by two liver surgeons and a corresponding anesthesiologist in Chang Gung Memorial Hospital, Keelung. The central venous catheter (CVP) and arterial catheter were inserted after anesthesia and connected to the FloTrac system. The Pringle maneuver was routinely used with Huang's loop (Huang et al., 2018) for inflow occlusion with 5-to-15 mins occlusion-releasing intervals. The demographic data of the patients are shown in Table 1. All the data were approved by the Institutional Review Board of the Chang Gung Memorial Hospital (IRB No: 202001650B0).

**Table 1:** Baseline demographic characteristics of 82 patients receiving laparoscopic hepatectomy.

**Characteristics**	**Variables**	**Values**
**Patient factors**		
Age (years)	Median (R)	69 (39–89)
Sex	Male (*n*, %)	47 (57.3%)
Child-Pugh classification	A (*n*, %)	82 (100%)
Etiology of liver tumor	HCC (*n*, %)	52 (63.4%)
	Cholangiocarcinoma (*n*, %)	12 (14.6%)
	Metastasis (*n*, %)	9 (11.0%)
	Benign (*n*, %)	9 (11.0%)
Viral hepatitis	HBV (*n*, %)	25 (30.5%)
	HCV (*n*, %)	17 (20.7%)
	None (*n*, %)	40(48.8%)
**Laboratory data**		
ICG15 (%)	>10 (*n*, %)	31 (37.8%)
ALT (U/L)	Median (R)	23.5.0 (1.0–217.0)
WBC (x10^9^/L)	Median (R)	9,1 (2.8–10.8)
Hb (g/dL)	Median (R)	13 (7.6–16.5)
Plt (x10^9^/L)	Median (R)	206.0 (103.0–639.0)
**Surgical factors**		
Surgical margin	R0 (*n*, %)	79 (96.3%)
Operation time (minutes)	>180 (*n*, %)	62 (75.6%)
EBL (cc)	>300 (*n*, %)	28 (34.1%)
RBC transfusion	N	9 (11.0%)
**Short term outcome**		
LOS (day)	Median (R)	6.0 (4.0–0.32.0)
Morbidity	*n* (%)	7 (9%)

AFP, alpha-fetoprotein; ALT, alanine transaminase; EBL, estimated blood loss; RBC, red blood cells; Hb, hemoglobin; Plt, platelets; HBV, hepatitis B virus; HCV, hepatis C virus; ICG15, indocyanine green retention rate at 15 mins; LOS, length of hospital stay; Median (R), median (range); WBC, white blood cells.

The FloTrac system continuously recorded the stroke volume variation (SVV), cardiac output (CO), systemic vascular resistance (SVR), and stroke volume resistance index (SVRI) every 20 secs, according to the manufacturer's manual, by connecting to the central venous catheter (CVC) and the arterial catheter. The start time and release time of the Pringle maneuver were also recorded in real-time. Perioperative demographic data were prospectively collected, including sex, age, virus infection, etiology of liver cancer, hemogram, coagulation function, renal function, and liver function.

We used growth mixture modeling (GMM) to analyze patient data, which extends the mixing effect model (MEM), also known as the latent class mixed model (LCMM), and is mainly used in longitudinal data studies. Based on the MEM and latent class analysis (LCA) modeling framework, the GMM with latent variables first divides the samples into potential groups and then applies an MEM model to each group to explain the differences between individuals within the group with respect to time. The comparison of the models is shown in the Supplementary material.

Through MEM, LCA, and GMM model discussion, we preprocessed the continuous data collected by the FloTrac system during the operation and used the GMM model to group and analyze the results. In addition, we also used latent class growth analysis (LCGA) to classify the model into groups. This model is a special case of the GMM model. The variance of the latent slope and intercept is fixed at zero within the class and is only allowed to vary between classes. Finally, conclusions were drawn from the analysis results. The flow chart of data extraction and analysis is shown in Figure 2B.

We used the Wilcoxon rank-sum test to verify whether there was a significant difference between the clusters. The Wilcoxon rank-sum test is a non-parametric method proposed by the American statistician Frank Wilcoxon, meaning that the test does not make any assumptions about the distribution of the underlying data. The Wilcoxon rank-sum test determines whether the median difference between two samples is greater than, less than, or equal to a specific value, and this test is equivalent to the Mann-Whitney U test.

## Results

In this study, 52 cases were hepatocellular carcinoma (63.4%) and male patients were more predominant (57.3%). Preoperative liver function was well preserved without advanced liver cirrhosis, and three-quarters of patients underwent an operation of more than 3 h. One-third of patients had blood loss of more than 300cc but only 11% required blood transfusion. Morbidity was noted in seven patients, but no patients had post-hepatectomy liver failure. The median postoperative hospital stay was 6 days. As shown in Table 2, the short-term laboratory data had significantly change at postoperative day one.

**Table 2:** Short-term physiological changes.

**Demographic**	**N (*n* = 82)**	**Mean (SD)**			
Age	82	67.43 (11.82)			
Height, cm	79	159.48 (8.87)			
Weight, kg	79	63.47 (12.56)			
**Variables**	**Before**		**After**		***P*** **value**
	**N (*****n*** = **82)**	**Mean (SD)**	**N (*****n*** = **82)**	**Mean (SD)**	
International Normalized Ratio (INR)	81	1.01 (0.06)	78	1.10 (0.11)	<0.0001
Aspartate aminotransferase (AST), U/L	79	33.59 (23.72)	81	337.56	<0.0001
Alanine aminotransferase (ALT), U/L	80	36.99 (44.72)	81	321.20	<0.0001
Total bilirubin, mg/dL	81	0.50 (0.36)	81	0.77 (0.47)	<0.0001
Direct bilirubin, mg/dL	45	0.26 (0.33)	79	0.62 (0.57)	<0.0001
Creatinine (Cr), mg/dL	80	1.17 (0.96)	81	1.38 (1.56)	0.1671
Segment (Seg), %	81	62.57 (9.86)	81	83.64 (6.42)	<0.0001
Lymph, %	81	27.49 (9.06)	81	10.19 (6.16)	<0.0001
White blood cells (WBC), 10^∧^3/uL	81	6.75 (1.89)	81	10.60 (2.90)	<0.0001
Platelets (PLT), 10^∧^3/uL	81	212.84 (78.89)	81	159.66 (57.14)	<0.0001
Blood Urea nitrogen (BUN), mg/dL	78	19.98 (10.50)	81	17.41 (13.70)	0.0185
Albumin, g/dL	75	4.31 (0.36)	73	3.64 (0.34)	<0.0001

^*^p, paired samples t-test was for means of continuous data between before and after.

**Figure 3:**
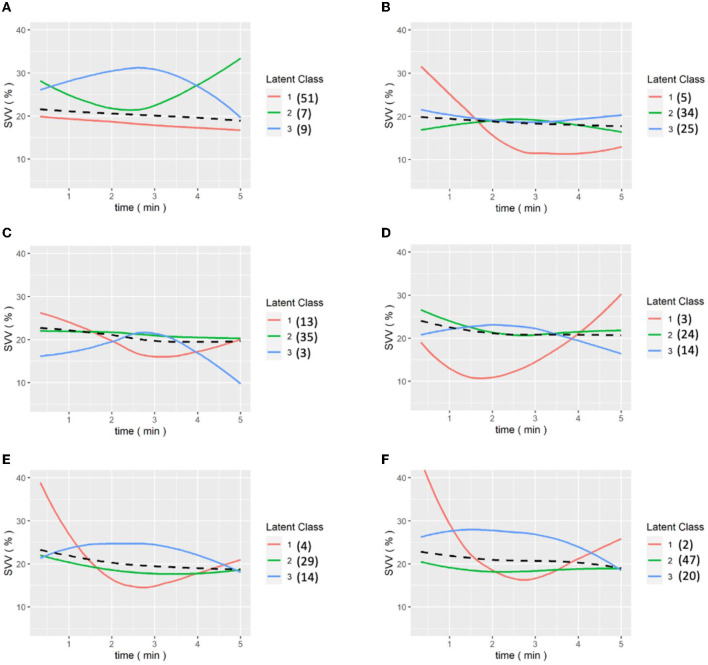
**(A–E)** are the grouping trajectories of SVV from the first release to the last release of Pringle maneuver, and **(F)** is the grouping trajectory of SVV int the last release of Pringle maneuver for each patient. The brackets represent the number of patients in each class.

**Figure 4:**
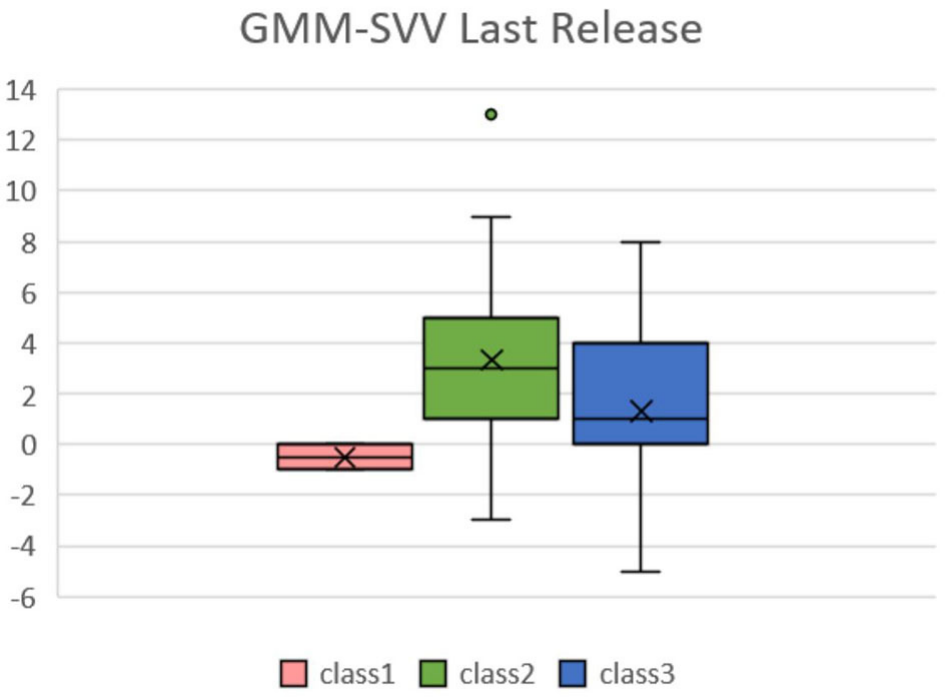
Box plots of SVV in the last release of Pringle maneuver relative to MELD-Na(delta) in the LCGA model. Patients in the second class have higher post-operative MELD-No compared with pre-operative data.

We then analyzed the data from the continuous data recorded by the FloTrac system and divided patients into three groups because the intraoperative conditions changes during the whole procedure. The amount of data collected was limited, so the classification results may be biased by extreme values, which is why we divided the patients into three classes to identify the outliers. Patients presented different responses after the release of the Pringle maneuver, which can ensure better venous return and decreased SVV. Figure 3 shows the GMM subgroup results for each release of Pringle maneuver. The black dashed line represents the MEM curve of the patients. The red, green, and blue curves represent latent classes 1, 2, and 3, respectively, and it can be observed that most of the patient subgroups are very similar to the MEM curves, which means that our model captures the overall patient profiles. In addition, the results of (a) to (f) show that there are only single-digit patients in a certain category, which indicates that the GMM model has classified the outliers. We found some correlation between SVV release and MELD-Na (delta). In order to have the same baseline for the patients, we compared the first and last clustering results for each patient. As shown in Figure 4, each color corresponds to the GMM cluster result. Because we used the median as the standard, we could draw a box plot with the MELD-Na (delta). The second class of patients with SVV Last Release had higher postoperative MELD-Na (delta) scores. To demonstrate whether there was a significant difference, the Wilcoxon rank-sum test was used. As shown in Table 3, the *p*-value of class 2 and class 3 in SVV Last Release was 0.0189, which means that the flatter the SVV value during the last release, the more significant the increase in MELD-Na score after surgery. In addition, the *p*-value of class1 and class 2 during the last release reached 0.0418, but because the number of patients in class 1 was too small, this was not discussed.

**Table 3:** Results of the Wilcoxon rank-sum test of CO and MELD-Na (delta) in the GMM model.

**GMM-SVV**				
		**Class 1**	**Class 2**	**Class 3**
First release	Class 1	–	–	–
	Class 2	0.5729	–	–
	Class 3	0.3608	0.4238	–
		Class 1	Class 2	Class 3
Last release	Class 1	–	–	–
	Class 2	0.0418	–	–
	Class 3	0.2241	**0.0189**	–

^*^The p-value of the last SVV release was 0.0418 (class 1 and class 2), but since there were only two patients in class 1, this was not discussed. Bold values indicate p <0.05.

In the intraoperative CO pringle, from a data perspective, the third pringle is more stable than the previous one. As shown in the figure below, the statistical analysis of the GMM at the time of CO Pringle reveals that, at the third Pringle maneuver, the grouping result of the patients gradually tended to stabilize, and the stable state meant that the CO index of the patients were within the normal range, 4 to 8 L/min.

**Figure 5:**
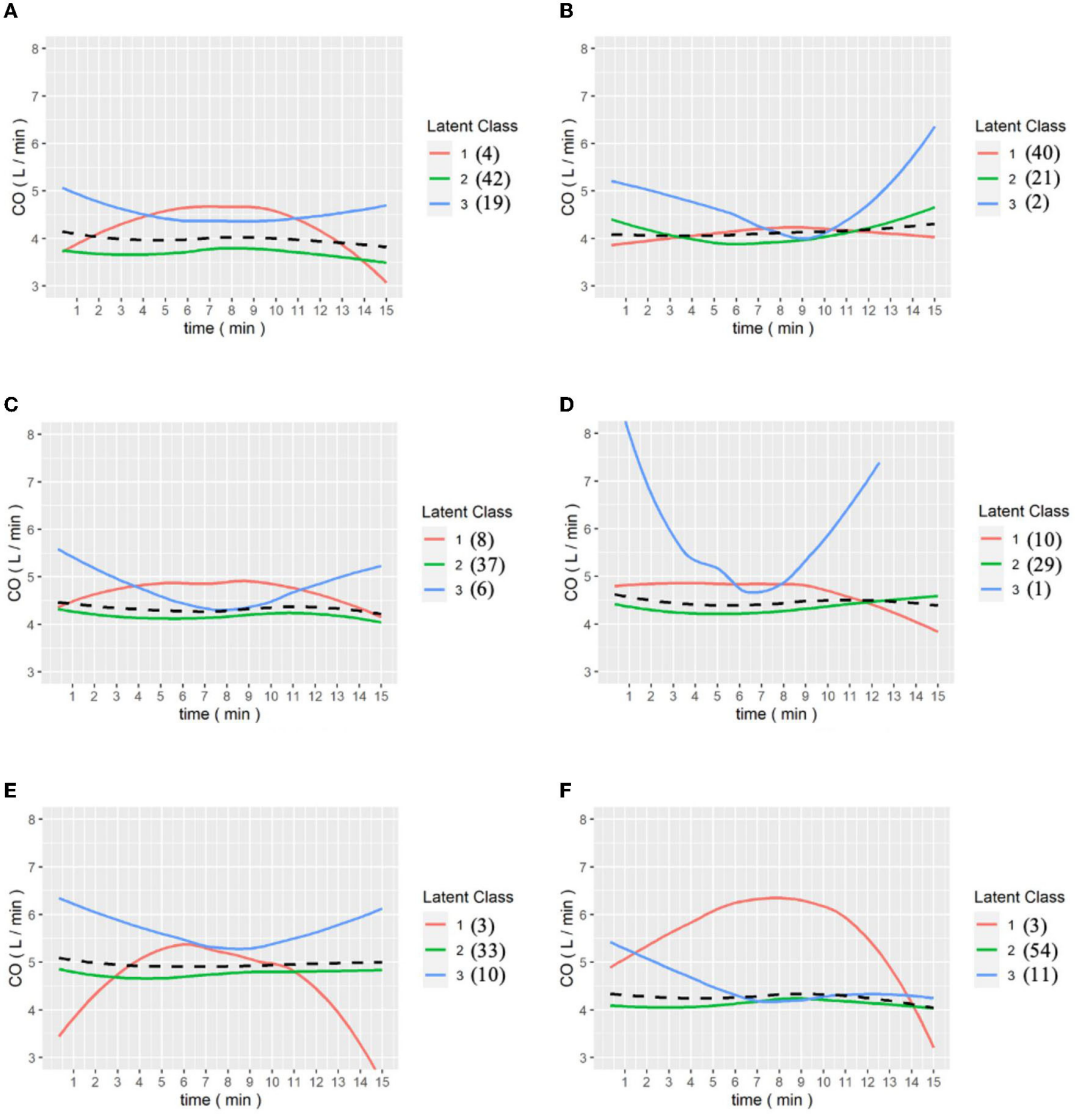
**(A–E)** are the grouping trajectories of Cardiac output(CO) from the first to the last Pringle maneuver, and **(F)** is the grouping trajectory of each patient's CO in the last Pringle maneuver. The brackets represent the number of patients in each class.

In particular, in Figure 5F, for the last Pringle maneuver of each patient, it can be observed that, except for a few outliers in the first cohort, all patients tended to approach a value of 4 L/min. This means patients' physiological functioning returned to normal after completion of the operation and resuscitation was initiated to restore the intravascular volume.

**Figure 6:**
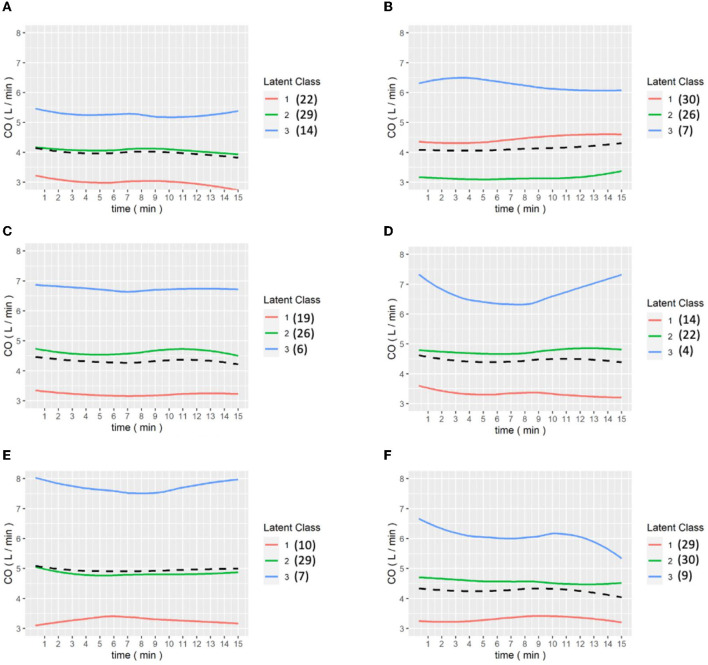
**(A–E)** are the grouping trajectories of CO from the first to the last Pringle maneuver in the LCGA model, and **(F)** is the grouping trajectory of each patient's CO in the last Pringle maneuver. The brackets represent the number of patients in each class.

**Figure 7:**
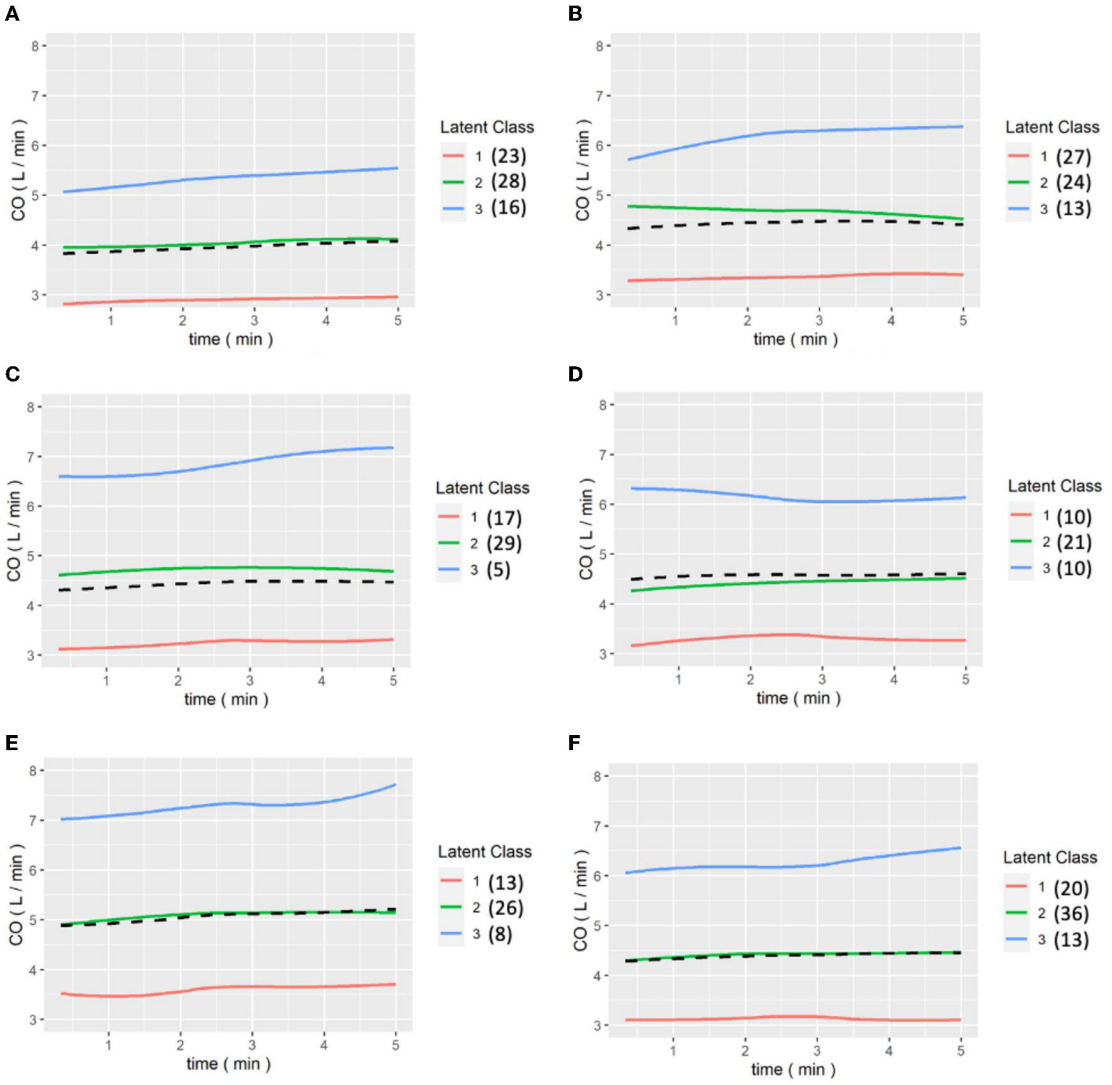
**(A–E)** are the grouping trajectories of CO from the first to the last release of Pringle maneuver in the LCGA model, and **(F)** is the grouping trajectory of each patient's CO in the last release of Pringle maneuver. The brackets represent the number of patients in each class.

Figures 6, 7 show the results of the LCGA model for the cohort of patients. It can be seen that the LCGA model is less prone to crossover than the GMM model and the three classes are all flat, with only high or low differences. In addition, the LCGA model has a more even number of patients in each class.

**Figure 8:**
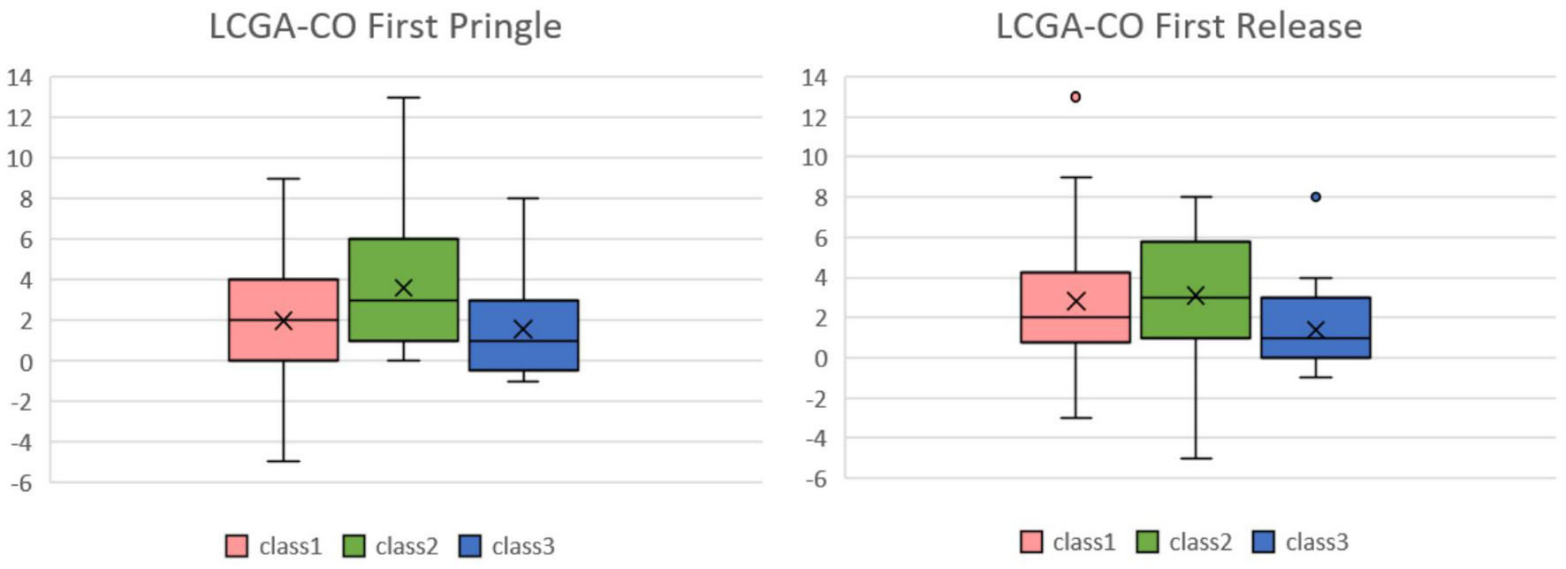
Box plots of CO in the first Pringle maneuver and CO in the first Release of Pringle maneuver relative to MELD-Na(delta) in the LCGA model. In the box plot of MELD-Na(delta) for each class of CO in the first Pringle maneuver and release in the LCGA model, the median MELD-Na(delta) of class 2 is higher than the other two classes. CO, cardiac output; MELD, model of end stage liver disease.

We also examined the LCGA model by following the GMM analysis. Figure 8 shows the box plot of MELD-Na (delta) for each class of CO in the first Pringle maneuver and release in the LCGA model; the median of class 2 was higher than the other two classes. The results are shown in Table 4. The *p*-value of class 2 and class 3 of CO in the first Pringle maneuver was 0.0299, which means that the MELD-Na (delta) of patients in class 2 was significantly higher than that of class 3.

**Table 4:** Results of the Wilcoxon rank-sum test of CO and MELD-Na (delta) in the LCGA model.

**LCGA-CO**				
		**Class 1**	**Class 2**	**Class 3**
First Pringle	Class 1	–	–	–
	Class 2	0.1848	–	–
	Class 3	0.4864	**0.0299**	–
		Class 1	Class 2	Class 3
First Release	Class 1	–	–	–
	Class 2	0.4261	–	–
	Class 3	0.1096	**0.032**	–

Bold values indicate p <0.05.

## Discussion

From our analysis of perioperative hemodynamic monitoring data, the response of rapid stroke volume variation to baseline data affected the short-term postoperative MELD-Na score. Patients with rapid recovery of the stroke volume had minimal changes of post-operative MELD-Na score, while patients whose stroke volume failed to recover after release of the Pringle maneuver had affected end-organ function and elevated MELD-Na scores. The Pringle maneuver decreased liver inflow and reduced blood loss effectively during hepatectomy. However, ischemic reperfusion injury (IRI) is critical in the inflammatory response, Kupffer cell activation, and cytokine release and is related to cellular damage (Cannistrà et al., 2016). The 50-50 criteria on postoperative day 5 is a wellvalidated predictor of postoperative liver failure, including the prolongation of prothrombin time (PT) <50% and elevation of serum bilirubin >50% (Balzan et al., 2005). The end stage liver disease (MELD) score model was first introduced in 2007 to predict the survival of patients with portal hypertension who underwent elective transjugular intrahepatic portosystemic shunts (Buell et al., 2009). Although the MELD score is not well accepted for predicting post-hepatectomy liver failure, it can quickly reflect the coordination and injury to liver and renal function after hepatectomy, either by IRI or low intravascular volume, and can be used in various perioperative prediction models to assess the perioperative risks (Pandey et al., 2012; Newman et al., 2020).

Linear regression is a commonly used type of predictive model, and it only considers fixed effects. If the data involve repeated measures and random effects, the between-groups and within-subjects factors should be further investigated (Han et al., 2012). The traditional mixed effect model (MEM) only considers heterogeneity between individuals, but heterogeneity between groups should also be considered when dealing with medical data. Latent class analysis (LCA), also known as the finite mixture model, assumes that there are latent subgroups in the data and aims to identify these latent subgroups based on the observed information (Clark and Muthén, 2009). It can be applied to many problems, including factor analysis, clustering, and regressions.

The growth mixture modeling (GMM) method used in this study extends the MEM with combination of LCA and has also been increasingly used in recent years (Herle et al., 2020), for example, in the social sciences to observe the association between longitudinal data on children's body mass indexes and parental perceptions of children's picky eating. Falkenstein et al. also applied this method to the treatment response of patients with obsessive-compulsive disorder (Falkenstein et al., 2019). However, even though these methods are increasingly being used, no one has performed a longitudinal data trajectory analysis on laparoscopic surgery, so we offer a method to identify and interpret these trajectories and suggest a relationship with postoperative indicators. Therefore, we used the GMM method to excavate the correlation of intraoperative dynamic physiological change and postoperative physiological reservation and can comprehensively evaluate the mixed effects of patient reservation, anesthesiology, intraoperative blood loss, fluid resuscitation, and maybe also the inotropic agents used to keep vital signs stable.

During the first Pringle maneuver, the patients seldom faced massive bleeding; the SVV response after the first Pringle maneuver is correlated with the physiological reserve of the individual patient. Patients without these responses implied an inadequate intravascular volume for end-organ perfusion. This could be an alarm signal for the surgical team to provide adequate volume expansion intraoperatively to avoiding postoperative liver decompensation instead of sticking to low CVP levels.

We also noted the SVV during the last Pringle cycle reflected the actual intravascular volume and affected the postoperative MELD-Na score. Our results showed that if the SVV did not change during the last release, the MELD-Na score increased during postoperative follow-up. Because our statistical methods could evaluate the mixed effects during the operation, the normalization of SVV in the last release of the Pringle maneuver reflected the condition of effective intravascular volume and can be used as a guide for postoperative resuscitation to prevent inadequate end-organ perfusion. We also analyzed the correlation of CO and MELD-Na, but its prediction value is not as the GMM model to analyze the change of SVV. The CO during the Pringle maneuver and release were stable without significant changes and patients with normal CO still had higher MELD-Na scores after the operation even compared with patients with lower intra-operative CO. We think that CO is a comprehensive physiological reflection after autoregulation and cannot be used as a real safety parameter for intra-operative monitoring. SVV should be prioritized for intra-operative fluid management in laparoscopic hepatectomy than CO from our observation.

The 50–50 criteria needs serial follow-up until postoperative day 5 to detect the risk of post-hepatectomy liver failure (Balzan et al., 2005). This study is a potential indicator of further fluid expansion even if hepatectomy has been completed and SVV can also be used to guide the volume required during postoperative resuscitation. As low CVP level and goal-directed fluid management are well accepted for perioperative care, fluid resuscitation after the operation may sometimes not be precisely according to different patients in different intravascular fluid situations. The response of cardiac output and end-organ perfusion would affect the postoperative MELD-Na score, therefore, the response at the reperfusion stage can help reflect the actual physiological reserve of the patient. Adequate resuscitation after surgery according to the change in SVV provides a potential preventive strategy in post-hepatectomy liver failure. During the operation, depth of anesthesia, intraoperative blood loss and volume expansion, and the inotropic agent used to meet the balance of a low CVP level and stable intraoperative hemodynamics made a complicated network effect. It would be hard work to calculate all the effects and draw all their weight; however, we only used the final comprehensive outcomes recorded by the FloTrac system to present the intraoperative changes. The response of our physiological change is dynamic and multifactorial and poor to predict or calculate due to the net-work effects, and we used the statistical method with a continuous physiological monitor to count both the fixed effects model and random effects model. A recent study revealed that the rapid turnover protein score before an operation could be correlated with oncological outcomes in HCC patients. This study revealed that the dynamic perioperative changes could affect the long-term outcome (Yanagaki et al., 2021). The previous study on simultaneous laparoscopic liver resections with colorectal cancer surgery achieved better oncological results than staged treatment of metastasis (Chen et al., 2019). Perioperative blood transfusions were also found to affect HCC recurrence during long-term follow-up (Harada et al., 2015). Our previous concept of the oncological outcome is related to the differentiation of the malignant tumor, the staging of the disease, and the definite treatment of the malignancy. However, perioperative dynamic changes should be given more attention in addition to traditional factors for the long-term oncological outcomes. This study provides a method for continuous intraoperative monitoring of vital signs that includes the comprehensive performance of physiological changes in each individual.

The FloTrac system has been widely used in perioperative fluid management and volume optimization (Elgendy et al., 2017). The application of SVV to replace central venous pressure (CVP) in hepatectomy was also well studied in perioperative management (Dunki-Jacobs et al., 2014). Current studies have mostly focused on intraoperative changes monitored by the FloTrac system, but our findings suggest that maintaining adequate stroke volume for end-organ perfusion during the re-perfusion stage is also critical for the perioperative outcomes.

### Clinical implication and suggestion for future practice

This study proved the concept that analyzing continuous intraoperative hemodynamic data using GMM is able to predict short-term postoperative physiological changes. It provides an alarm system and the chance for preemptive volume expansion if the recovery of stroke volume variation declines, potentially increasing the safety of laparoscopic hepatectomies.

## Limitations

Although the sample size was small, the results were consistent with the statistical model, which can be extended to more patients and provide a more powerful prediction value. More randomized studies are essential for further investigation.

## Conclusion

The complexity of the hemodynamic data recorded by the FloTrac system during the Pringle maneuver in laparoscopic hepatectomies can be effectively analyzed using the GMM method. The results can potentially predict the risk of short-term liver function deterioration.

## Data availability statement

The original contributions presented in the study are included in the article/Supplementary material, further inquiries can be directed to the corresponding author.

## Ethics statement

The studies involving human participants were reviewed and approved by Institutional Review Board of the Chang Gung Memorial Hospital. The patients/participants provided their written informed consent to participate in this study.

## Author contributions

Y-CC, M-HL, and R-SS wrote the manuscript. R-SS and C-LL designed the research. M-HL and S-NH performed the research. M-HL, S-NH, and C-KH analyzed the data. All authors contributed to the article and approved the submitted version.
