# Supplementary material for: The influence of the Pringle maneuver in laparoscopic hepatectomy: continuous monitor of hemodynamic change can predict the perioperatively physiological reservation
Source: Front Big Data. 2023 Jun 14;6:1042516. doi: 10.3389/fdata.2023.1042516 (PMC10303928; doi:10.3389/fdata.2023.1042516)
Supplement: Supplementary file 1 [file Table_1.DOCX]

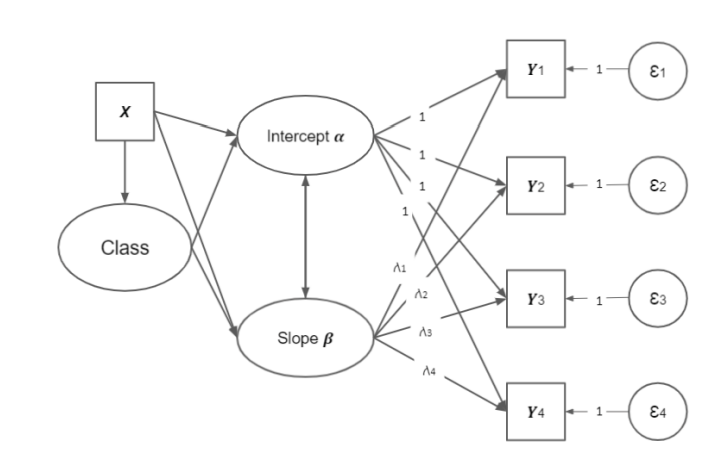


Structural equation modelling representation of GMM

The main difference between GMM and MEM is that GMM can take into account the heterogeneity of intercept and slope in the MEM model and add the latent class for grouping the samples into clusters.

Comparison of MEM ,LCA, GMM and LCGA model

| Method | Description |
| --- | --- |
| Mixed Effects Model (MEM) | The MEM model not only extends the linear regression model but also takes into account the heterogeneity between individuals. " Mixed " means that the model contains both a fixed effect ($\beta_{0}$ , $\beta_{1}$) and a random effect ($b_{0i}$ , $b_{1i}$) |
| Latent Class Analysis (LCA) | It is a method of finding and measuring unobserved latent subgroups based on the response to a set of observed variables. It can be applied to factors, clusters and regressions. |
| Growth Mixture Model (GMM) | It is also known as Latent Class Mixed Model (LCMM), and is mainly used in longitudinal data studies.  Based on the modeling framework of MEM and LCA,the GMM with latent variables first divides the sample into potential groups, and then applies the MEM model to each group to explain the differences of the individuals within the group with respect to time. The main difference of GMM is that it can take into account the heterogeneity of intercept and slope in the MEM model and add the latent class for grouping the samples into clusters. |
| The Latent Class Growth Model (LCGA) | LCGA is a special case of GMM in that the variance of the potential slope and intercept is fixed to zero within a class and only allowed to vary between classes.  LCGA assumes that the growth trajectory of all individuals within a category is homogeneous. This method may be particularly useful when faced with small sample sizes or other statistical problems such as failure due to overly complex models that do not converge. |

We use R version 4.0.5 and package hlem{lcmm} version 1.9.2 to develop the models. This revision has provided the parameter settings used by the models in the manuscript, as shown below.

| Parameter | Values | Description |
| --- | --- | --- |
| fixed | X0~1+time+I(time^2) | Two-sided linear formula object for the fixed-effects in the linear mixed model. |
| mixture | ~1+time+I(time^2) | One-sided formula object for the class-specific fixed effects in the linear mixed model |
| random | ~1+time | Optional one-sided formula for the random-effects in the linear mixed model |
| ng | 3 | Optional number of latent classes considered |
| nwg | FALSE | Optional logic indicating if the variance-covariance of the random-effects is class-specific.  If FALSE the variance-covariance matrix is common over latent classes. |
